# Improving osteoarthritis management in primary healthcare: results from a quasi-experimental study

**DOI:** 10.1186/s12891-021-03959-6

**Published:** 2021-01-14

**Authors:** Nina Østerås, Irma Brandeggen Blaker, Tore Hjortland, Elizabeth Cottrell, Jonathan G. Quicke, Krysia S. Dziedzic, Steven Blackburn, Aksel Paulsen

**Affiliations:** 1 Division of Rheumatology and Research, National Advisory Unit on Rehabilitation in Rheumatology, Diakonhjemmet Hospital, Oslo, Norway; 2 Hillevåg Fysioterapi og Trening, Stavanger, Norway; 3 Legesenteret Mariero, Stavanger, Norway; 4 Impact Accelerator Unit, Versus Arthritis Primary Care Centre, Keele University, Staffordshire, ST5 5BG UK; 5 Department of Orthopaedics, Division of Surgery, Stavanger University Hospital, Stavanger, Norway; 6 Department of Public Health, The Faculty of Health Sciences, University of Stavanger, Stavanger, Norway

**Keywords:** Osteoarthritis, Implementation, Quality of care

## Abstract

**Background:**

To improve quality of care for patients with hip and knee osteoarthritis (OA), general practitioners (GPs) and physiotherapists (PTs) in a Norwegian municipality initiated an intervention. The intervention aimed to increase provision of core OA treatment (information, exercise, and weight control) prior to referral for surgery, rational use of imaging for assessing OA and improve communication between healthcare professionals. This study assessed the effectiveness of this intervention.

**Methods:**

Forty-eight PTs and one hundred one GPs were invited to the intervention that included two interactive workshops outlining best practice and an accompanying template for PT discharge reports. Using interrupted time series research design, the study period was divided into three: pre-implementation, transition (implementation) and post-implementation. Comparing the change between pre- and post-implementation, the primary outcome was patient-reported quality of OA care measured with the OsteoArthritis Quality Indicator questionnaire. Secondary outcomes were number of PT discharge reports, information included in GP referral letters to orthopaedic surgeon, the proportion of GP referral letters indicating use of core treatment, and the use of imaging within OA assessment. Analyses involved linear mixed and logistic regression models.

**Results:**

The PT workshop had 30 attendees, and 31 PTs and 33 GPs attended the multidisciplinary workshop. Two hundred eight and one hundred twenty-five patients completed the questionnaire during pre- and post-implementation, respectively. The adjusted model showed a small, statistically non-significant, increase in mean total score for quality of OA care (mean change = 4.96, 95% CI -0.18, 10.12, p:0.057), which was mainly related to items on OA core treatment. Patients had higher odds of reporting receipt of information on treatment alternatives (odds ratio (OR) 1.9, 95% CI 1.08, 3.24) and on self-management (OR 2.4, 95% CI 1.33, 4.32) in the post-implementation phase. There was a small, statistically non-significant, increase in the proportion of GP referral letters indicating prior use of core treatment modalities. There were negligible changes in the number of PT discharge reports, in the information included in the GP referral letters, and in the use of imaging for OA assessment.

**Conclusion:**

This study suggests that a primary care intervention including two inter-active workshops can shift the quality of care towards best practice recommendations.

**Trial registration:**

ClinicalTrials.gov: NCT02876120.

**Supplementary Information:**

The online version contains supplementary material available at 10.1186/s12891-021-03959-6.

## Background

Osteoarthritis (OA) is a serious joint disease characterized by pain, disability, and impaired quality of life. Prevalence of OA increases with age, and nearly one in two people will develop symptomatic knee OA and one in four symptomatic hip OA in their lifetime [1, 2]. With an aging population and the epidemic of obesity, the prevalence of OA is set to rise [3]. OA is one of the leading causes of pain and disability for the adult population worldwide [4] and one of the major contributors to years lived with disability [5]. The costs of treatment and work-related losses represent a considerable economic burden [6, 7].

Recommended first-line, core treatments include patient education, self-management, exercise, and weight reduction [4, 8–10]. These core interventions can reduce pain and improve function and should be offered to all individuals with symptomatic OA. Joint replacement offers an effective option for those with pain and reduced function that has a substantial impact on their quality of life and are refractory to non-surgical treatment [11]. However, it is costly and associated with medical and surgical risks [12–15], and although most patients benefit greatly from the operation [16–18], it is not always effective [19]. One in ten hip and one in five knee joint replacements have painful joints postoperatively [20]. Due to the increasing prevalence of OA, the demand for joint replacements is expected to accelerate and quadruple by 2030 [21]. Since it is estimated that only 12–53% of patients with symptomatic OA should be offered arthroplasties [22], it is important to improve the uptake of high-quality non-surgical care. In addition, previous research has suggested an overuse of resource intensive Magnetic Resonance Imaging (MRI) in the diagnosis and treatment of moderate to severe OA [23]. Since decisions on joint replacement can be made using the less resource intensive conventional radiographs, and use of Magnetic Resonance Imaging (MRI) is usually unnecessary [24], this resource overuse should be addressed.

Evidence based recommendations and standards of care for OA management have existed, and remained consistent, for over a decade [4, 8–10], but provided care often does not align well with recommended treatment modalities [25–27]. This is particularly evident for the core treatments. Among physiotherapists’ (PTs) practice, exercise treatment is frequently provided, but PTs also tend to provide several other treatment modalities with no evidence or moderate to low quality of evidence (e.g., acupuncture, electrotherapy) [28–31]. In order to provide best value OA care, it is important to both implement evidence based cost-effective care and reduce the use of care with no or limited evidence and at the same time rationalize resource use (e.g., use group-based treatments). Previous research indicate that both general practitioners (GPs) and PTs are reluctant to discuss weight issues with their patients [32–34]. It has also been shown that GPs favoured monitoring patients’ physical function, pain and analgesia use over body mass index (BMI), self- management plans and exercise advice [35]. Indeed, some GPs feel they have insufficient expertise to advise patients about exercise [36]. Further, few Norwegian PTs send discharge reports routinely [37], which may limit the GPs’ knowledge on effects of physical therapy and restrain the communication between health professionals.

A small number of best practice initiatives to improve quality of OA care have been conducted with diverging results [38–44]. The current study, the STavanger osteoARThritis (START) study, was initiated by a group of clinicians (GP, PT, and orthopaedic surgeon) working in primary and specialist healthcare services. The purpose of START was to employ an intervention to increase the communication between GPs and PTs, to improve the quality of OA care by implementing evidence-based treatment recommendations for OA care, and to evaluate the impact of the intervention on: 1) alignment of care with guideline recommendations, 2) frequency of PTs providing discharge reports to the GPs through provision of a template for discharge report, 3) frequency of GP referral letters to orthopaedic surgeons including pre-defined relevant information, 4) frequency of GP referral letters indicating prior use of core treatment, and 5) frequency of GP referral letters indicating that the patient had an MRI taken, and not a conventional radiograph, for decision making about joint replacement surgery.

## Methods

### Design and setting

The START study, undertaken between September 2016 and November 2017, employed an interrupted time series design, which is considered to be the strongest quasi-experimental research design [45]. The study was conducted in Stavanger Municipality, Norway, which has a population of approximately 130,000 inhabitants. It represents a collaborative study between GPs and PTs in Stavanger primary healthcare, an orthopaedic surgeon at the Department of orthopaedics at Stavanger University Hospital, and researchers at National Advisory Unit on Rehabilitation in Rheumatology at Diakonhjemmet Hospital, Oslo. The START study was prospectively registered at clinicaltrials.gov (NCT02876120) and is reported according to the TREND checklist [46]. The START study was linked to the “Joint Implementation of Guidelines for oSteoArthritis in Western Europe” (JIGSAW-E) implementation approach (https://jigsaw-e.com/) which was based upon the MOSAICS study [47] where implementation science and knowledge mobilisation theory (e.g. Normalisation Process Theory, implementation theory, the Theoretical Domains Framework and principles of adult learning) was used to guide implementation.

**Fig. 1:**
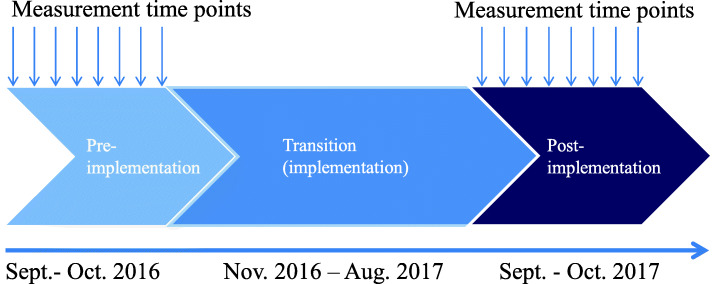
Study timeline

The START study was divided into three periods: pre-implementation, transition (implementation) and post-implementation period (Fig. 1). The data collection took place during pre- and post-implementation periods, with each lasting 8 weeks. The intervention was implemented during the transition period, which lasted 44 weeks. The transition period was planned to be long to give the healthcare professionals time to implement the intervention and change their practice. This way, the data collection in the pre- and post-implementation periods took place at the same time of the year (autumn).

The Regional Committee for Medical and Health Research Ethics (Ref. no.: 2016/1734 REK South-East D) and The Data Inspectorate/ Data Protection Official at Stavanger University Hospital (Ref. no.: 2016/31) approved the study.

### Participants

All GPs and PTs working in private practice in Stavanger Municipality who saw adults with OA were invited to participate in the study. The GPs received a written invitation from the Stavanger Municipality Chief Medical Officer to attend a multidisciplinary workshop. Two of the PTs in the project group visited all PT clinics and invited the PTs to attend the PT workshop and the multidisciplinary workshop. Patient participants were recruited by the PTs during the pre- and the post-implementation periods. The pre- and the post-implementation participants were different patients. All patients that had received physiotherapy treatment for at least 2 weeks (4–6 sessions) due to symptomatic hip and/or knee OA fulfilled the inclusion criterion. All patients with language- or other impairments disqualifying completion of patient-reported quality of OA care questionnaire were excluded.

### Blinding

The GPs, PTs and patient participants were informed on the purpose of the study, but they remained blinded regarding the study outcome measures.

### Intervention

**Fig. 2:**
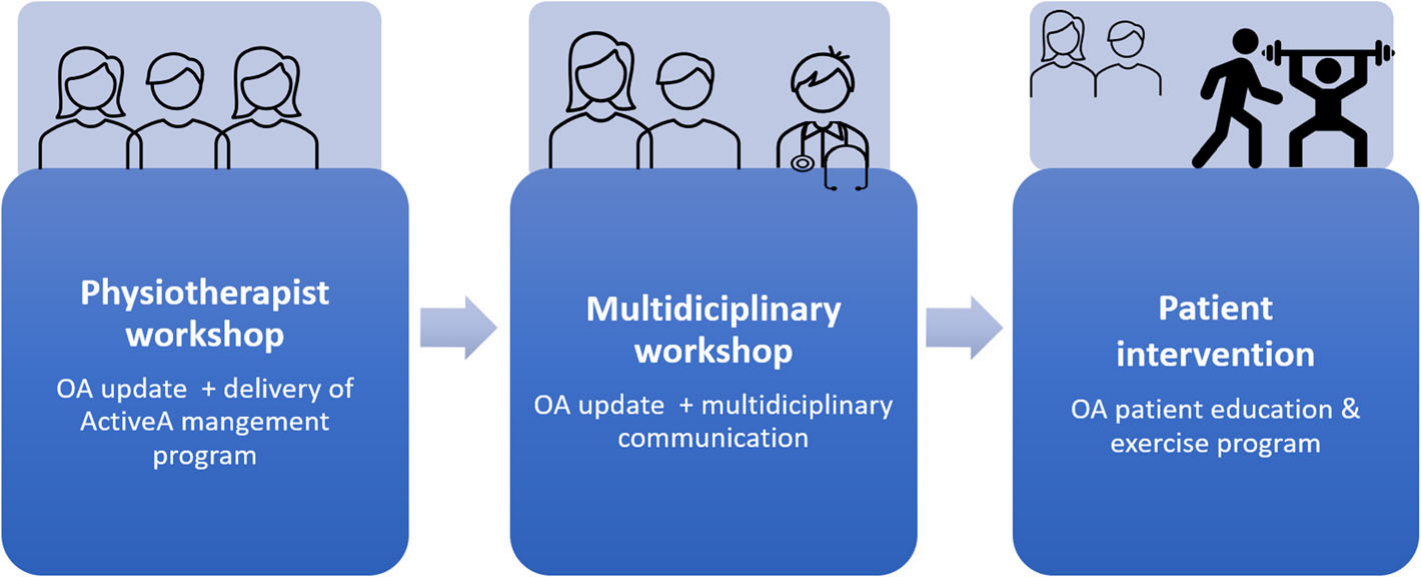
Overview of the intervention components

The intervention intended to facilitate communication between health professionals in primary healthcare and with healthcare professionals in specialist healthcare to ensure that people with hip and knee OA would experience timely, well integrated, and high-quality OA care (Fig. 2). The intervention included a PT workshop and a multidisciplinary workshop and was linked to the JIGSAW-E project (https://jigsaw-e.com/) and inspired by the SAMBA study [48] . The aim of JIGSAW-E was the implementation of an approach to improve the quality of care and to support self-management of OA in primary care. The JIGSAW-E approach includes four innovations: 1) an OA guidebook written by patients and health professionals, 2) a model consultation for diagnostic and follow-up consultations, 3) training for health professionals delivering the care, and 4) quality indicator recording and measurement tools. The SAMBA study intervention included a PT workshop and a multidisciplinary workshop to implement a model for structured OA care in primary health care. Compared to the JIGSAW-E and the SAMBA interventions, the START study included some new elements: distribution of a template for PT discharge reports and a discussion on indications for joint replacement, and a focus on the content of GP referral letters to orthopaedic surgeons.

### The PT workshop

To learn about current OA treatment recommendations, eligible PTs were encouraged to attend the PT workshop arranged in Stavanger on the 26th of October 2016. The workshop included a one-day (9 h) OA educational programme organised by the “Active with osteoArthritis” (ActiveA) management programme [36], which builds on similar Swedish [41] and Danish [42] programmes. The workshop included an update on OA epidemiology, clinical features, and treatment recommendations. Education in delivery of a patient OA education programme, individually tailored semi-standardised exercises, performance testing, and healthy eating and weight reduction strategies was given. The PTs received access to the ready-to-use patient OA education programme (PowerPoint file and manuscript) and access to a database with recommendations for resistance exercises and dose specifications. As part of the ActiveA management programme, the PTs were encouraged to regularly arrange group-based 3 h patient OA education programmes followed by a 6 weeks exercise programme with twice weekly 1-h supervised group sessions (For more details, see Østerås et al. [49]).

### The multidisciplinary workshop

The local project group (AP, HW, IBB and TH) arranged one multidisciplinary workshop in each of the six town districts. The separation by districts was done purposely to facilitate communication between GPs and PTs working in the neighbourhood. The second purpose of the workshop was to present current recommendations for OA treatment. The 1.5-h multidisciplinary workshop took place after work hours during November and December 2016, which was at the start of the transition period. It included an update on current treatment recommendations for OA care and a presentation of the PT-led patient OA education and exercise programme (the ActiveA programme). An orthopaedic surgeon presented views on when to consider referral to secondary care, relevant information to include in the GP referral letter, and recommendations for the use of imaging modalities for decision making about joint replacement surgery. Towards the end of the workshop, the local project group facilitated a multidisciplinary discussion regarding OA management.

### Development of a PT discharge report template

Research on barriers or facilitators for writing discharge reports are lacking, but the project group hypothesized that time could be a limiting factor and that PTs’ uncertainty regarding appropriate content and quantity could represent barriers. To standardize the content and potentially reduce the time needed for PTs to write discharge reports, a template for OA discharge reports was developed by the project group and distributed to the PTs. The one-paged template included the following items: patient characteristics, treatment period, pre-treatment assessment (4–5 lines), treatment goal, treatment effect conclusions (3–4 lines), and post-treatment assessment (including pain level at activity/rest/night, restricted joint range of motion, muscle strength, joint stability, physical function, walking ability and walking aids, physical performance tests, OA-specific patient reported outcomes, work participation, smoking and body weight/body mass index).

### Data collection

The data collection took place during two periods of 8 weeks: the pre-implementation period on September 19th -November 6th in 2016 and the post-implementation period on September 18th -November 5th in 2017.

The patient participants self-reported on an anonymous two-paged paper questionnaire handed out by the PTs after a treatment session and put it in a sealed envelope before leaving the clinic. The questionnaire included the following items: age, sex, OA joint location (hip/knee, uni. vs. bilateral), joint prosthesis in hip/knee (yes/no; uni. vs. bilateral), pain level (Likert scale, five levels), physical function (Likert scale, five levels) and patient-reported quality of OA care (quality indicators (QIs), 16 items).

Number of discharge reports to referring GPs was extracted from the Norwegian Health Economics Administration (Helfo), which is the Directorate of Health’s external agency responsible for making payments from the National Insurance scheme to healthcare providers, suppliers and service providers, as well as individual refunds of expenses.GP referral letters were extracted from the Stavanger University Hospital’s register.

### Primary outcome

The primary outcome was change in patient-reported quality of OA care from pre- to post-implementation period. It was measured with the OsteoArthritis Quality Indicator questionnaire version 2 (OA-QI v2) (Additional file 1) [50]. OA-QI v1 was developed in 2010 based on published QIs for OA care identified in a literature search and was refined using expert panels and patient interviews [51]. A minor revision was undertaken in 2015 [50]. OA-QI v2 reflects current OA care guideline recommendations [4, 8–10] and includes 16 QI items related to patient OA education and information, regular provider assessments, referrals, and pharmacological treatment. Previous applications of the questionnaire have showed acceptable measurement properties including reliability, validity, responsiveness, and interpretability [50, 51]. OA-QI v1 has been previously tested in UK primary care in a cluster-randomised trial and has been shown to be responsive to the use of national recommendations for OA care [43].

An example of an item with response alternatives is as follows: ‘Have you been given information about osteoarthritis from a health professional? Yes/No/Don’t remember’. Each QI item was considered passed if the patient had checked ‘Yes’ and was considered ‘eligible’ if the patient responded ‘Yes’ or ‘No’ for that item. On the patient level, the QI pass rate was calculated as the total number of items passed divided by the number of eligible items for each patient (in percentage), ranging from 0 to 100, with 100 representing the best quality of care score. On the group level, the mean total pass rate was calculated.

### Secondary outcome measures

#### PT discharge reports

The number of Stavanger PTs’ registered discharge reports to referring GPs was extracted from the Helfo register. The content could not be accessed, but the number of discharge reports was seen as a quality indicator given the low level of reports in traditional care.

#### Information in GP referral letters

Stavanger GPs’ referral letters to the Department of Orthopaedic Surgery at Stavanger University Hospital during pre- and post-implementation periods were anonymized by a secretary outside the project group. Referrals related to acute conditions (e.g., ligament ruptures, infections), hip impingement conditions or post-operative review were excluded. The project group discussed and agreed beforehand on a list of 31 items that they considered relevant to be included in GP referral letters. The list was based on current guideline recommendations (e.g., that core treatment should be provided prior to surgery) and other information that the orthopaedic surgeons need for their evaluation of whether the patient is a candidate for arthroplasty. Two of these items (medication list and comorbidity) are normally automatically included in the electronic referral. Inclusion of information in the anonymized referral letters was scored (included vs. not included) by the first author.

### Core treatment

The proportions of people with OA that, according to the GP referral letter, had used one or more of the core treatment modalities (physiotherapy, supervised exercise, weight reduction, OA information and/or OA education programme) prior to orthopaedic referral, were calculated.

#### Use of imaging for OA assessment

The proportion of people that, according to the GP referral letter, had been assessed using an MRI for decision making on joint replacement surgery, but not a conventional radiograph, were calculated. Those with no use of imaging were ignored.

Deviation from the protocol (clinicaltrials.gov: NCT02876120): We were unable to assess the proportion of people with OA referred to orthopaedic surgeon in secondary care that underwent scheduled joint surgery. This was due to not being able to determine relevant patient cases within the hospital’s ICT system.

### Statistical analyses

The patient samples represent convenience samples. A time series-repeated observations of outcome measurements was collected during the pre- and post-implementation with the 8 + 8 weeks representing 16 time points. Linear mixed models showed no slopes within the pre- or the post-implementation period. It was therefore decided to pool data for the 8 time points during the pre- and the 8 time points in the post-implementation period. A linear multi-level mixed models with random intercepts was fitted to adjust for the effect of clustering (PT clinic). There were 1–7 PTs working in each PT clinic. Since the intraclass correlation coefficient (ICC) was very low (< 0.001) and single-level and multi-level analyses gave similar results, a multilevel model was considered unnecessary. Hence, the primary outcome was assessed applying a single-level model with a before-after design to evaluate level changes from the pre-implementation period to the post-implementation period. The final regression model was adjusted for patients’ age and sex. Individual OA-QI v2 items were analyzed applying logistic regression crude models and the models were adjusted for patients’ age and sex.

The proportions of referral letters with vs. without relevant information on each of the 31 predefined items were assessed with unadjusted logistic regression models, or with unadjusted logistic regression with the Firth procedure for bias reduction in rare events. To account for multiple analyses testing, a Bonferroni correction of the *p*-value was done (*p* = 0.0016). A randomized subsample (20%) of the GP referral letters was re-scored by a co-author (AP), and the two sets of scores were compared. Inter-rater reliability for the two raters (NØ and AP) was examined by calculation of perfect agreement and kappa. Statistical analyses were performed using STATA/IC 14.

### Patient and public involvement and engagement

Two patient research partners were involved during study planning and in development of study materials (including the questionnaire and patient information). Using the GRIPP2 reporting checklist [52], a summary of the patient and public involvement and engagement in the study is presented in the Additional file 2.

## Results

In 2016, 101 GPs and 48 PTs in private practice in Stavanger were eligible for the START study. Thirty PTs in Stavanger attended the ActiveA workshop in October 2016. Another 10 PTs had previously attended an ActiveA workshop (prior to the transition period), and one PT clinic (involving 2 PTs) had started providing OA education and exercise programme to their patients prior to the transition period. Thirty-one (65%) of the PTs and 33 (33%) of the GPs attended the multidisciplinary workshop. In total 13 of the 15 eligible PT clinics and 22 of the 31 GP practices within Stavanger were represented at the workshop.

During the pre-implementation period, 208 patient participants were recruited from 13 PT clinics, and 125 patient participants from 11 PT clinics were recruited during the post-implementation period (Table 1). The number of patients per clinic ranged from one to 57 during pre-implementation and two to 30 during post-implementation periods. The patient participants’ mean age was 63 years at pre- and 65 years at post-implementation, and females accounted for 72 and 65% of the two samples, respectively. Patients’ characteristics are provided in Table 1.

**Table 1:** Patient characteristics in pre- and post-implementation samples

	Patient participants	
Variable	pre-implementation (***n*** = 208)	post-implementation (***n*** = 125)
Sex, female, n (%)	150 (72)	81 (65)
Age, mean (SD)	63 (10)	65 (10)
Osteoarthritis joint location, n (%)		
Hip/hips	48 (24)	33 (27)
Knee/knees	75 (37)	47 (39)
Hip and knee and/or multisite	81 (40)	42 (34)
Hip or knee joint prosthesis, n (%)		
No joint prosthesis	157 (75)	92 (74)
One joint	35 (17)	25 (20)
Two or more joints	16 (8)	8 (6)
Pain, n (%)		
No pain	1 (0.5)	4 (3)
Very mild pain	18 (9)	13 (10)
Mild pain	44 (21)	22 (18)
Moderate pain	112 (54)	71 (57)
Severe pain	28 (14)	15 (12)
Self-reported function, n (%)		
Very good function	3 (1)	2 (2)
Good function	57 (27)	36 (29)
Neither good nor poor function	67 (32)	47 (38)
Poor function	70 (34)	32 (26)
Very poor function	6 (3)	8 (6)

The hospital received 104 GP referral letters for assessment by an orthopaedic surgeon during the pre-implementation period, 86 of these were included in this evaluation and 18 were excluded (not OA-related condition *n* = 7, post-operative review *n* = 6, acute condition *n* = 5). For the post-implementation period, the hospital received 114 referral letters, of which 103 were included and 11 were excluded (acute condition *n* = 6, not OA-related condition *n* = 2, hip impingement *n* = 1, post-operative review *n* = 1, referred by hospital doctor *n* = 1).

### Primary outcome

**Fig. 3:**
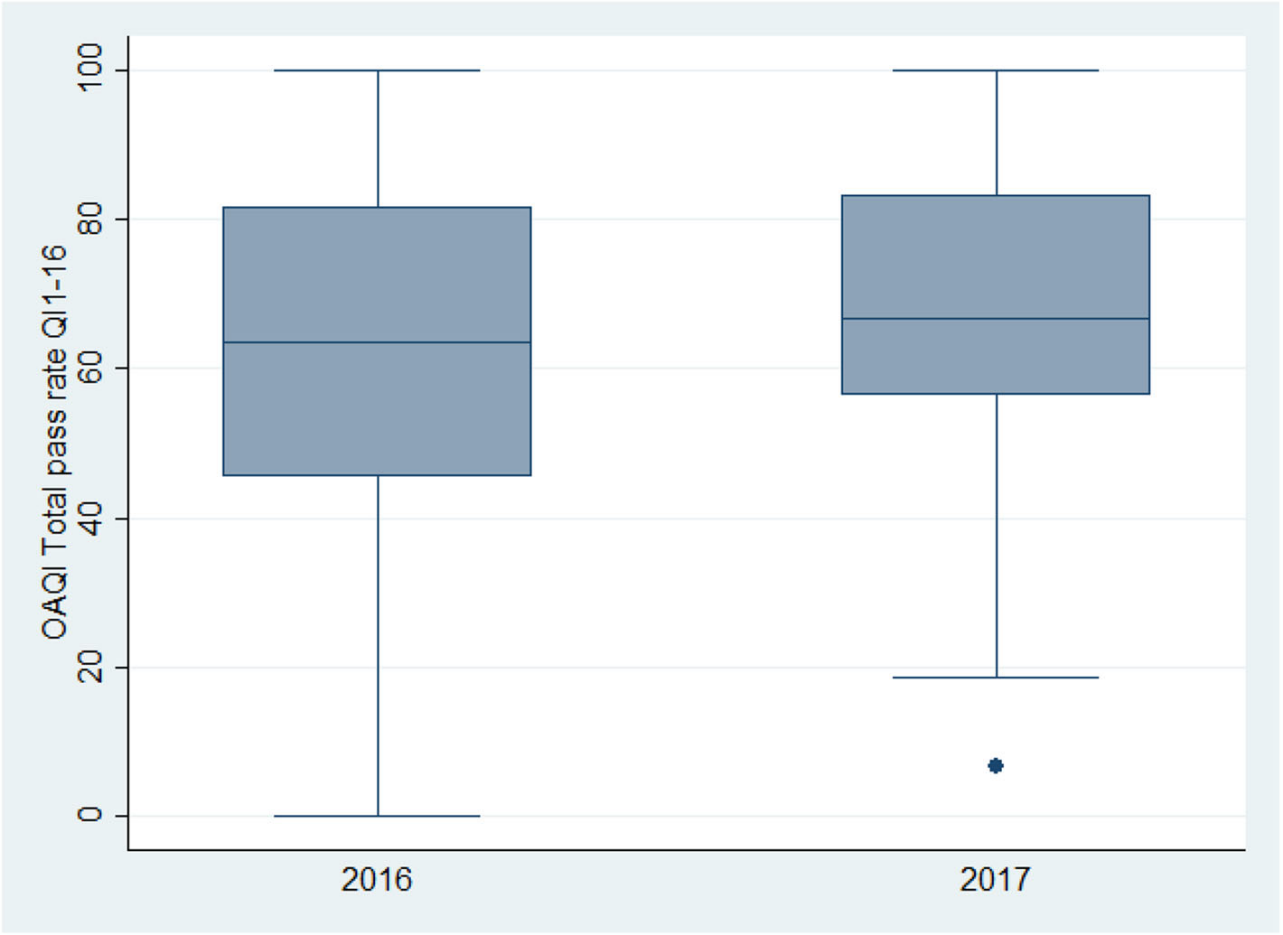
Patient-reported quality of osteoarthritis care. Boxplot of total mean score during the pre-implementation in 2016 (*n* = 208 patient responders) and the post-implementation in 2017 (*n* = 125 patient responders)

**Fig. 4:**
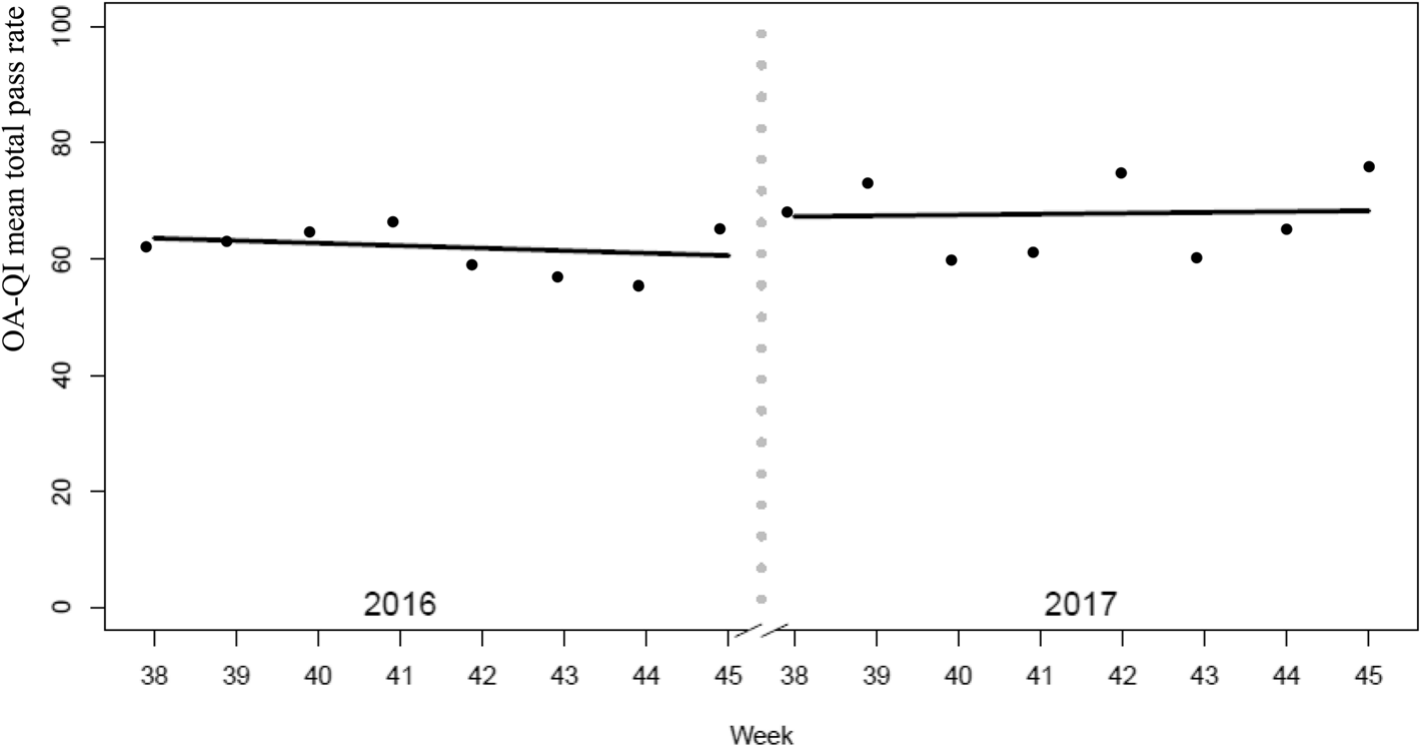
Patient-reported quality of osteoarthritis care. Marginsplot of total mean score during 8 pre-implementation weeks in 2016 (*n* = 208 patient responders) and 8 post-implementation weeks in 2017 (*n* = 125 patient responders)

The OA-QI v2 mean (SD) total pass rate increased from 62.4 (23.6) to 67.7 (21.2) from pre-to post-implementation period (mean difference 5.3; 95% confidence interval (CI) 0.31, 10.35; *p* = 0.037) as illustrated in Fig. 3. When adjusted for patient age and sex, the difference became statistically non-significant (mean difference 4.96; 95% CI -0.18, 10.12; *p* = 0.057). Figure 4 illustrates that there was no slope during any of the periods, but that there was a small level change from the pre- to post-implementation period.

**Fig. 5:**
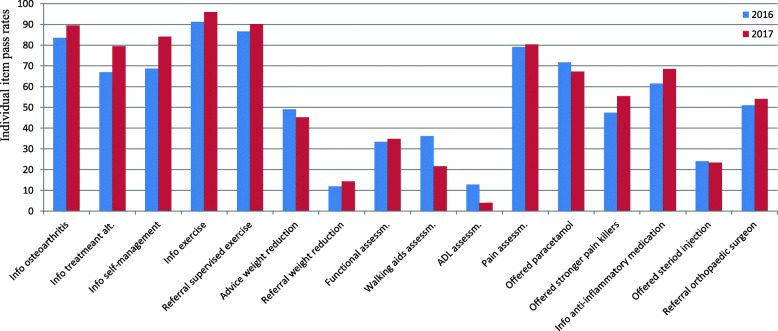
Patient-reported quality of osteoarthritis care: pass rates for individual OAQI v2 items during pre-implementation (*n* = 208) and post-implementation (*n* = 125). All items have ‘Yes’, ‘No’, and ‘Not applicable’/‘Don’t remember’ as response options. Individual item pass rates were calculated as the proportion of patients reporting that the QI was passed (‘Yes’) divided by the proportion of patients who were eligible (‘Yes’ or ‘No’) for that QI item (in percentage). Pass rates range from 0 to 100, with 100 representing that all eligible patients reported pass (‘Yes’) for that QI item. OAQI v2 items wording: 1) Have you been given information about osteoarthritis from a health professional? 2) Have you been given information about different treatment alternatives? 3) Have you been given information about how you can self-manage the disease? 4) Have you been given information about the importance of physical activity and exercise? 5) Have you been referred or offered a referral to a health professional who can advise you about physical activity and exercise? 6) Have you been advised to lose weight if you are overweight? 7) Have you been referred or offered a referral to someone who can help you to lose weight, if you are overweight?8) If you have problems with daily activities, have these problems been assessed by a health professional? 9) If you have problems with walking, has your need for a walking aid been assessed? (e.g. stick, crutch or walker) 10) If you have problems related to other daily activities, has your need for appliances and aids been assessed? (e.g. splints, assistive technology for cooking or personal hygiene, a special chair) 11) If you have joint pain, has it been assessed by a health professional? 12) If you have joint pain, was paracetamol the first medication that was recommended? 13) If you have prolonged severe joint pain, which is not relieved sufficiently by paracetamol, have you been offered stronger pain killing medications? (e.g. co-codamol, codeine, tramadol, co-proxamol, co-dydramol, dihydrocodeine) 14) If you use anti-inflammatory medications, have you been given information about the effects and possible side-effects of this medication? (e.g. ibuprofen (Nurofen, Brufen), diclofenac (Voltarol), naproxen (Naprosyn), celecoxib (Celebrex)) 15) If you have experienced an acute deterioration of your symptoms, have you been given or offered a steroid injection? 16) If you are severely troubled by your osteoarthritis, and exercise and medication do not help, have you been referred or offered a referral for an assessment for operation? (e.g. joint replacement)

Individual QI item pass rates are provided in Table 2. Figure 5 illustrates the differences in item pass rates during pre- and post-implementation periods. Eight individual item pass rates increased from pre- to post-implementation by 3–12 percentage points, four were mainly unchanged and four showed a reduction in pass rates by 4–14 percentage points. The pass rates increased for all five items on information: about the OA disease, treatment alternatives, self-management, importance of physical activity and on potential effects and side effects of anti-inflammatory medication. The patient participants had significantly higher odds during post-implementation for having received information on different treatment alternatives (Odds ratio (OR) 1.9; 95% CI 1.08, 3.24; *p* = 0.024) and on self-management (OR 2.4; 95% CI 1.33, 4.32; *p* = 0.003) compared to during pre-implementation. Higher pass rates post- compared to pre-implementation were seen for most of items reflecting recommended core treatment, but not for the two items regarding weight management, for which the pass rate was unchanged or slightly reduced.

**Table 2:** Patient-reported quality of osteoarthritis care: individual item pass rates and odds ratio for item achievement

Individual QI items	Individual item pass rates^a^			OR (95% CI) for item achievement			
	Pre-implemen-tation	Post-implemen-tation	Δ Post – Pre pass rates	Crude model	***p***-value	Adjusted model^b^	***p***-value
Information about OA from a HP (*n* = 308^c^)	84	89	↑5	1.7 (0.83, 3.40)	0.152	1.8 (0.86, 3.60)	0.124
Information about different treatment alternatives (*n* = 308)	67	79	↑12	**1.9 (1.08, 3.24)**	**0.024**	**1.8 (1.04, 3.14)**	**0.037**
Information on self-management (*n* = 308)	69	84	↑15	**2.4 (1.33, 4.32)**	**0.003**	**2.6 (1.43, 4.79)**	**0.002**
Information about importance of physical activity (*n* = 328)	91	96	↑5	2.2 (0.81, 6.19)	0.120	2.3 (0.83, 6.47)	0.108
Referred to HP for physical ctivity/exercise (*n* = 316)	87	90	↑3	1.4 (0.68, 2.93)	0.344	1.4 (0.66, 2.86)	0.406
Advised to lose weight (*n* = 158)	49	45	↓4	0.9 (0.44, 1.66)	0.649	0.8 (0.43, 1.65)	0.618
Referred for support to lose weight (*n* = 163)	12	14	↓2	1.2 (0.45, 3.31)	0.680	1.0 (0.33, 2.87)	0.966
Assessed for functional ability (*n* = 186)	33	35	↑2	1.1 (0.57, 2.01)	0.835	1.1 (0.56, 2.06)	0.823
Assessed the need for walking aids (*n* = 168)	36	22	↓14	0.5 (0.24, 1.01)	0.055	0.5 (0.25, 1.10)	0.089
Assessed the need for other aids (*n* = 151)	13	4	↓9	0.3 (0.06, 1.30)	0.105	0.3 (0.06, 1.32)	0.109
Joint pain assessed by HP (*n* = 309)	79	80	↑1	1.1 (0.61, 1.91)	0.804	1.1 (0.62, 1.97)	0.746
Paracetamol recommended as first line (*n* = 303)	72	67	↓5	0.8 (0.49, 1.34)	0.415	0.9 (0.53, 1.48)	0.639
Offered stronger pain killers (*n* = 213)	47	55	↑8	1.4 (0.78, 2.42)	0.271	1.3 (0.75, 2.38)	0.319
Information about anti-inflammatory medication (*n* = 206)	62	68	↑6	1.4 (0.74, 2.47)	0.321	1.3 (0.70, 2.37)	0.412
Offered steroid injection (*n* = 206)	24	23	↓4	1.0 (0.50, 1.87)	0.915	0.9 (0.47, 1.83)	0.837
Referred to orthopaedic surgeon (*n* = 208)	51	54	↑3	1.1 (0.64, 2.02)	0.648	1.1 (0.64, 2.04)	0.658

*HP* Healthcare professional, *OA* Osteoarthritis, *OR* Odds ratio, *QI* Quality indicator

^a^ Individual item pass rates were calculated as the proportion of patients reporting that the QI was passed (‘Yes’) divided by the proportion of patients who were eligible (‘Yes’ or ‘No’) for that QI item (in percentage). Pass rates range from 0 to 100, with 100 representing that all eligible patients reported pass (‘Yes’) for that QI item

^b^ Logistic regression model adjusted for patients’ sex and age

^c^ Number of eligible patients for the QI item, i.e., patients that have responded ‘Yes’ or ‘No’ to that item. Patient responses ‘Not applicable’ are ignored

### Secondary outcomes

#### PT discharge reports

Both the total number of discharge reports and number of PTs writing discharge reports showed a small increase from pre- to post-implementation. The number of registered PT discharge reports was 149 (registered by 24 PTs) during the pre- and 158 (registered by 30 PTs) during the post-implementation period. The mean (min-max) discharge reports per PT was 6 (1–27) during pre- and 6 (1–21) during post-implementation.

#### Information in GP referral letters

Overall, only minor changes in the information included in GP referral letters were observed from pre- to post-implementation period (Table 3). However, there was a clear increase in the proportion of referral letters that included information on duration of pain symptoms and smoking cessation from pre- to post-implementation (Table 3).

**Table 3:** Information in GPs’ referral letters of patients to orthopaedic surgeon during pre- and post-implementation

	% referrals that included this information during			
Items considered relevant to be included in GPs’ referral letters: Information on …	pre-implementation (***n*** = 86)	post-implementation (***n*** = 103)	OR (95% CI) for reporting this information	***p***-value^a^
Results from a clinical examination	45	51	1.2 (0.69, 2.18)	0.482
Restricted joint range of motion	35	30	0.8 (0.43, 1.48)	0.484
Joint malalignment	7	11	1.6 (0.56, 4.51)	0.379
Reduced joint stability	9	12	1.3 (0.50, 3.31)	0.602
Skin conditions, if relevant for potential surgery^b^	0	3	6.0 (0.31, 118.3)	0.111
Affection of walking ability	64	53	0.6 (0.35, 1.12)	0.111
Eventual use of walking aids^b^	6	6	1.0 (0.31, 3.19)	0.997
The activity level	55	53	1.0 (0.54, 1.69)	0.863
Health related quality of life	10	8	0.7 (0.27, 1.96)	0.519
The pain level	92	80	0.3 (0.11, 0.76)	0.012
The duration of pain symptoms	**53**	**81**	**3.6 (1.89, 6.89)**	**< 0.001**
Eventual progression of pain	51	70	2.2 (1.22, 4.03)	0.009
Night pain	44	46	1.0 (0.60, 1.89)	0.842
Conventional radiograph (CR) being taken	57	57	1.0 (0.57, 1.81)	0.966
Magnetic resonance image (MRI) being taken	47	49	1.1 (0.61, 1.93)	0.781
The CR and MRI image(s) being recent^c^	84	84	1.0 (0.45, 2.13)	0.967
Used physiotherapy	22	31	1.2 (0.65, 2.33)	0.515
Used supervised exercise	14	21	1.7 (0.77, 3.62)	0.190
Having received information on the OA disease and treatment alternatives^b^	2	4	1.5 (0.32, 7.37)	0.597
Having participated in an OA education programme^b^	1	4	2.6 (0.40, 16.7)	0.321
Paracetamol use	41	45	1.2 (0.66, 2.10)	0.584
NSAID use	62	56	0.8 (0.45, 1,44)	0.460
Topical NSAID use^b^	5	2	0.5 (0.09, 2.18)	0.322
Opioid use	35	27	0.7 (0.34, 1.30)	0.253
Used non-surgical treatment alternatives for three to six months	13	15	1.2 (0.50, 2.68)	0.725
Overweight or body mass index	12	9	0.7 (0.28, 1.88)	0.512
Having tried weight reduction, if relevant^b^	6	1	0.2 (0.03, 1.35)	0.101
Smoking status	22	7	0.3 (0.12, 0.72)	0.007
Having tried to stop smoking, if relevant^b^	7	2	**0.1 (0.03, 0.41)**	**0.001**
Comorbidity	86	76	0.5 (0.24, 1.08)	0.078
Current medications	91	86	0.7 (0.26, 1.64	0.362

*HP* Health professional, *NSAIDS* Non-Steroidal Antiinflammatory Drugs, *OA* Osteoarthritis, *OA-QI v2* OsteoArthritis Quality Indicator questionnaire version 2, *QI* Quality indicator

^a^ To account for multiple analyses testing, a Bonferroni correction of the p-value was done (*p* = 0.0016)

^b^ Analyzed using logistic regression with the Firth procedure for bias reduction in rare events

^c^ Recent was defined as within the past 12 months

#### Core treatment

According to the GP referral letters, there was a small, but statistically non-significant, increase in the proportion of people with OA that had exploited the core treatment modalities before being referred to orthopaedic surgeon (31% vs. 34%, OR 1.2; 95% CI 0.61, 2.07; *p* = 0.706). The proportion of referral letters including information on prior use of physiotherapy increased from 22 to 31%, but the difference in odds did not reach statistical significance (OR 1.2; 95% CI 0.65, 2.33; *p* = 0.515).

#### MRI and conventional radiographs for OA assessment

The proportions that had taken an MRI for OA assessment were 47% (*n* = 40) during pre- and 49% (*n* = 50) during post-implementation period. Among these, the proportions that had only taken MRI, and not conventional radiographs, were similar during the pre- and post-implementation periods: 63% (25/40) vs. 64% (32/50), respectively.

#### Inter-rater reliability

On the referral level, the inter-rater reliability was acceptable with a perfect agreement mean score of 94% (min-max: 84–100%). On the single item level, inter-rater reliability was acceptable with a perfect agreement mean score of 96% (min-max: 68–100%). The item ‘Information on pain level included’ showed the lowest agreement, since one rater scored ‘Yes’ and the other rater scored ‘No’ on six (32%) of the 19 referral letters. For all other items, the agreement was very high. The mean kappa was 0.77 with values ranging from 0.44 to 1.00.

## Discussion

The START study showed that after implementing international guidelines for OA care among PTs and GPs in primary healthcare, OA management indicated a non-significant, small shift towards improved alignment with international recommendations. The improvement was mainly related to core treatment modalities and was in particular evident for provision of information on OA treatment alternatives and on self-management. This was supported by a small, statistically non-significant increase in the proportion of GP referral letters to orthopaedic surgeons indicating that core treatment modalities had been previously used. However, patient-reported receipt of advice about weight management did not improve, and there were negligible to no changes related to PT discharge reports, information in GP referral letters, and the use of different imaging modalities for OA assessment.

The patient sample characteristics in this and previous studies [31, 48–51, 53–55] were comparable on age, sex and OA location, but the START study included a higher proportion of patient participants with joint prosthesis as compared with a previous implementation study [49]). The mean total pass rate for the pre-implementation period in the START study was much higher compared to other studies that have used the OA-QI questionnaire [31, 48–51, 53–55]. The high pre-implementation score in the START study may be related to differences in patient recruitment since this study recruited patient participants that had received 4–6 physiotherapy treatment sessions. This in contrast to the previous studies, in which patients were recruited from the general population [51, 55], GP practices [49, 53], at first session of physiotherapy [49], scheduled for a consultation with an orthopaedic surgeon [31], or among members of a patient organisation [54, 55]. In addition, 10 physiotherapists in this study had attended an ActiveA workshop prior to the transition phase, and 1 clinic had started providing OA education and exercise programme to their patients. This may also potentially have contributed to the high pre-implementation pass rate in the START study.

The 5 percentage points increase in mean total pass rate from the pre- to post-implementation period in the START study indicates that the provided care became somewhat more in line with the guideline recommendations. This finding was supported by the small increase in the proportion of GP referral letters indicating that core treatment had been exploited before surgical treatment options were considered. In the SAMBA study, a similar OA guideline implementation study, the mean total pass rate in the intervention group increased from 39% at baseline to 60% at the six-month follow-up [49]. Hence, the pre-implementation pass rate in the START study (62%) was higher than the post-implementation pass rate in the SAMBA study, which leaves a smaller scope of improvement in the START study. While the 5-point increase was higher than the previously reported measurement error of 3 for this instrument [50], the importance of a 5-point increase on the OA-QI questionnaire may be debated.

For individual OA-QI items post-implementation pass rates of 79–96% were seen for most core treatment items. These pass rates are comparable to the post-implementation rates for the intervention group in the SAMBA study [49] but are much higher than the pass rates in the observational studies [31, 48, 50, 51, 53–55]. This demonstrates the potential of judicious implementation studies in making OA management more in line with international treatment recommendations. However, the post-implementation pass rates for weight loss advice and for referrals to support for losing weight remained low in the START study as in the SAMBA study [49]. Also, observational studies have revealed low pass rates for weight management uncovering a large scope for improvement [31, 51, 53–55]. The low pass rates may be related to health professionals’ barriers towards discussing weight issues and their prioritizing to review other aspects of health [32, 33, 35]. Further, there are relatively few weight loss services in Norway that the GP can refer to, and the GPs may not be aware of those. In a previous study, a large proportion of PTs reported that they would address weight by the provision of advice, but some lacked confidence in addressing weight loss [33]. While PTs believe they have a role in addressing weight loss, they felt inadequately equipped to integrate weight loss in their management approach [56]. Since weight reduction has been shown to significantly reduce symptoms and improve function among those with overweight [57, 58], future studies should investigate how weight loss management for people with OA can be improved.

To our knowledge, only one small previous study has assessed Norwegians PTs’ practice of writing discharge reports [37]. As PT discharge reports may provide important information for the GP to review together with the patient, the discharge report may represent an element in the continuity of patient information transfer. Information from the PT to the GP on the effect of core treatment and the patients’ status may lead to more appropriate referrals for candidates for joint replacement and reduce the number of inappropriate referrals. In this study, the results showed only a small increase in the total number of registered discharge reports and in the number of PTs writing one or more discharge reports. While this study was a first attempt to make interprofessional communication a standard practice, ensuring the quality of the reports should be the next step in a future study. The results also revealed a large inter-provider variation with the number of discharge reports registered per PT ranging from none to more than 20. Hence, there may be other unknown barriers that hinder the PTs in writing discharge reports. It was out of the scope of this study to investigate other barriers or facilitators for writing discharge reports.

A previous study found that the content of GP referral letters to orthopaedic surgeons often was suboptimal [59]. This finding is supported in more recent studies on referral letters in other medical disciplines showing that basic items necessary for appropriate triage often were lacking [60, 61]. According to a Cochrane Review [62], active, local educational interventions including secondary care specialists are shown to impact referral practice, but this was not seen in the START study as there were very few changes in the content of referral letters or in the use of imaging modalities for OA assessment. The multidisciplinary workshop in the START study lasted only 1.5 h, and the information and discussion on information in referral letters and the recommendations for imaging may have fallen behind the other topics covered. A more intense focus in the workshop or provision of a checklist for recommended content may have improved the content of GPs’ referral letters.

Although a large proportion of the GP referral letters contained information on pain, only 8% included information on whether the patients’ quality of life was affected and barely half included information from a medical examination. Only a small proportion provided information on whether core and supplementary treatment options (e.g. medication) had already been tried. According to the NICE guideline [4], referrals for consideration of joint surgery should be restricted to people, in which joint symptoms have a substantial impact on their quality of life and the symptoms are refractory to non-surgical treatment. The results in this study indicate a large scope for improving the content of the referral letters to facilitate timely access to orthopaedic surgeons for consideration of joint replacement specialty care.

One strength of the START study was its alignment to the “Joint Implementation of Guidelines for oSteoArthritis in Western Europe” (JIGSAW-E) (https://jigsaw-e.com/) which was based upon the MOSAICS study [47] where implementation science and knowledge mobilisation theory (e.g. Normalisation Process Theory, implementation theory, the Theoretical Domains Framework and principles of adult learning) was used to guide implementation and intervention design. Another strength of this study is the use of opinion leaders/clinical champions as moderators in the multidisciplinary workshops and facilitators for the implementation of OA guidelines. The involvement of both primary and secondary healthcare services in this study emphasized the importance of continuity of patient care. The multidisciplinary workshops mobilized a large proportion of GPs and PTs in the Stavanger Municipality to become updated on OA management. The active involvement of patient research partners in the planning phase ensured relevant patient information and survey questions as well as advices regarding the data collection and patient confidentiality.

Some methodological limitations should be taken into consideration when interpreting the results of the present study. In light of recent research on sample size and power with an interrupted time series design [63], the small patient sample size in the START study indicates that the power may have been low. The long transition period means that only persistent effects were captured and that the effect of the intervention may have been underestimated. Further, that 2 PTs already were providing OA education and exercise in line with the ActiveA management programme during pre-implementation period, may have reduced the effect of the implementation in the START study. It may also be a limitation that we were unable to adjust for PT clustering of patients because the patient reports were anonymous. However, as the intervention was designed to standardize PTs’ approach and the ICC for the PT clinic level was very low, we do not expect a high variation between individual PTs’ approach in this study. That two PT clinics did not recruit patients post-implementation may be due to reduced engagement. When excluding patient responses from these two clinics in a sensitivity analysis, the CIs became slightly more narrow, but this did not change the conclusions. Whilst this is a pragmatic implementation study the pre-implementation patient participant number did drop notably to the post-implementation, which may reflect reduced engagement among the PTs, but a selection bias related to PTs’ in handing out the questionnaires cannot be ruled out. Since the number of eligible OA patients is unknown, we cannot calculate a response rate, and a patient selection bias cannot be ruled out. This also has consequences for the external validity. It is also a limitation that the registered PT discharge reports may have been related to patients with other diagnoses than OA. Since the GP referral letters were anonymized, it was not possible to distinguish between the referral letters from GPs that had attended the workshop versus from GPs that did not attend. Given that only 33% of the GPs attended the workshop, the impact of the workshop on all referral letters generated in the locality may have been diluted. To get very significant clinical outcomes, a huge and lengthy study would be required. A mixed methods approach may have provided more in-depth understanding, but the funding available did not permit the addition of a mixed methods quantitate and qualitative agenda. However, we believe this study adds important knowledge about where the gaps currently exist.

One clinical implication of this study is that a relatively small intervention can slightly shift clinicians’ provision of OA care to better align with guideline recommendations for the provision of patient information and recommending core OA treatment modalities. Future studies may need to be larger, include more multifaceted and targeted interventions, and also other outcome measurements, including qualitative process evaluations, in order to see effects related to other aspects of OA care (e.g. weight reduction advising, healthy lifestyle changes, changes in the written communication between health professionals, and in the use of imaging modalities for assessment of OA). In addition, future studies should explore how to widen health professionals’ engagement and to ensure persistence of behaviour change for health professionals as well as patients.

## Conclusions

Implementing international OA treatment recommendations in a primary healthcare setting is a grand endeavour, which normally requires multidimensional interventions. In this study implementing OA treatment recommendations in a primary healthcare setting through two inter-active workshops and implementation of a discharge report template, OA management showed small, non-significant changes towards better alignment with best practice treatment recommendations.

## Supplementary Information

**Additional file 1.** OsteoArthritis Quality Indicator questionnaire version 2 (OA-QI v2).

**Additional file 2.** GRIPP2-SF checklist.

## Data Availability

The datasets used and/or analysed during the current study are available from the corresponding author on reasonable request.

## Abbreviations

CI: Confidence interval
HELFO: Norwegian Health Economics Administration
OA: Osteoarthritis
GP: General practitioner
MRI: Magnetic resonance imaging
OR: Odds ratio
PT: Physiotherapist
