# Supplementary material for: Improving osteoarthritis management in primary healthcare: results from a quasi-experimental study
Source: BMC Musculoskelet Disord. 2021 Jan 14;22:79. doi: 10.1186/s12891-021-03959-6 (PMC7807869; doi:10.1186/s12891-021-03959-6)
Supplement: Supplementary file 1 — Additional file 1. OsteoArthritis Quality Indicator questionnaire version 2 (OA-QI v2). [file 12891_2021_3959_MOESM1_ESM.docx]

Additional file 1. OsteoArthritis Quality Indicator questionnaire version 2 (OA-QI v2)

| **Questions on the treatment of your osteoarthritis** | | | | | | | |  |
| --- | --- | --- | --- | --- | --- | --- | --- | --- |
| There are several different treatment alternatives for osteoarthritis. What treatment, information or advice have you received from health professionals for your osteoarthritis in the past __________? For each question, please cross off one of the boxes provided.  **Yes**  **No**  **Don't remember** | | | | | | | |  |
| 1 | Have you been given information about osteoarthritis from a health professional? | □ | | □ | | □ | |  |
| 2 | Have you been given information about different treatment alternatives? | □ | | □ | | □ | |  |
| 3 | Have you been given information about how you can self-manage the disease? | □ | | □ | | □ | |  |
| 4 | Have you been given information about the importance of physical activity and exercise? | □ | | □ | | □ | |  |
| 5 | Have you been referred or offered a referral to a health professional who can advise you about physical activity and exercise? | □ | | □ | | □ | |  |
|  | | **Yes** | | **No** | | **Not overweight** | |  |
| 6 | Have you been advised to lose weight, if you are overweight? | □ | | □ | | □ | |  |
| 7 | Have you been referred or offered a referral to someone who can help you to lose weight, if you are overweight? | □ | | □ | | □ | |  |
|  | | **Yes** | | **No** | | **No such problems** | |  |
| 8 | If you have problems with daily activities, have these problems been assessed by a health professional? | □ | | □ | | □ | |  |
| 9 | If you have problems with walking, has your need for a walking aid been assessed? (e.g. stick, crutch or walker) | □ | | □ | | □ | |  |
| 10 | If you have problems related to other daily activities, has your need for appliances and aids been assessed? (e.g. splints, assistive technology for cooking or personal hygiene, a special chair) | □ | | □ | | □ | |  |
|  | | **Yes** | | **No** | | **No pain** | |  |
| 11 | If you have joint pain, has it been assessed by a health professional? | □ | | □ | | □ | |  |
| 12 | If you have joint pain, was paracetamol the first medication that was recommended? | □ | | □ | | □ | |  |
|  |  | **Yes** | | **No** | | **No prolonged severe pain** | |  |
| 13 | If you have prolonged severe joint pain, which is not relieved sufficiently by paracetamol, have you been offered stronger pain killing medications? (e.g. co-codamol, codeine, tramadol, co-proxamol, co-dydramol, dihydrocodeine) | □ | | □ | | □ | |  |
|  |  | **Yes** | | **No** | | **Not taking such drugs** | |  |
| 14 | If you use anti-inflammatory medications, have you been given information about the effects and possible side-effects of this medication? (e.g. ibuprofen (Nurofen, Brufen), diclofenac (Voltarol), naproxen (Naprosyn), celecoxib (Celebrex)) | □ | | □ | | □ | |  |
|  |  | **Yes** | | **No** | | **Not experienced such deterioration** | |  |
| 15 | If you have experienced an acute deterioration of your symptoms, have you been given or offered a steroid injection? | □ | | □ | | □ | |  |
|  | | **Yes** | | **No** | | **Not severely troubled** | |  |
| 16 | If you are severely troubled by your osteoarthritis, and exercise and medication do not help, have you been referred or offered a referral for an assessment for operation? (e.g. joint replacement) | | □ | | □ | | □ | |
