# Supplementary material for: Improving osteoarthritis management in primary healthcare: results from a quasi-experimental study
Source: BMC Musculoskelet Disord. 2021 Jan 14;22:79. doi: 10.1186/s12891-021-03959-6 (PMC7807869; doi:10.1186/s12891-021-03959-6)
Supplement: Supplementary file 2 — Additional file 2. GRIPP2-SF checklist. [file 12891_2021_3959_MOESM2_ESM.docx]

Additional file 2. GRIPP2-SF checklist

| **Section, topic and item** | **Description** |
| --- | --- |
| **1. Aim:** Report the aim of PPI in the study | To work with clinicians and researchers as part of the START project group to implement OA treatment recommendations in on Norwegian municipality. |
| **2. Methods:** Provide a clear description of the methods used for PPI in the study | Two patient research partners were recruited through the two physiotherapists in the project group. Both  patient research partners had lived experience of OA. The patient research partners were involved from the early phases of the project during the planning of the START study. The project leader in the START study was responsible for patient research partner involvement.  The patients research partners were involved in regular meetings with project group and contributed by email and telephone for the review of documents and resources. The patient research partners were actively involved in many aspects of the project, including:   1. Decision-making and collaboration within the START project group to shape the implementation of core treatment for OA 2. Developing and refining innovations and patient-facing resources   One patient research partner also attended a JIGSAW-E Community of Practice meetings in the UK, where representatives from each of the five European countries convened to share knowledge and learn from each other’s experience of implementing the OA innovations. During this meeting, dedicated sessions were held for the patient research partners to network with each other, share experiences and discuss the role of patient and public involvement and engagement in the JIGSAW-E project. |
| **3. Study results:** Outcomes—Report the results of PPI in the study, including both positive and negative outcomes | Through lived experience of OA and knowledge of local OA provision, the patient research partners had an active role in shaping and adapting the START study innovations and patient information resources so that they were understandable, appropriate, and accessible. Specific contributions included:   - **Critically reviewing different Norwegian OA patient guidebooks:** The patient research partners were involved in reviewing different, available Norwegian OA patient guidebooks to decide which one that should be used in the START study intervention. The ActiveA patient OA guidebook scored highest among the patient research partners and was therefore used in this study. - **Translation and cultural adaption of one chapter of the JIGSAW-E OA Guidebook**   The patient research partners participated in translating and adapting one chapter of the JIGSAW-E OA Guidebook, chapter no. 6 “Feeling positive”, that could compliment the ActiveA OA guidebook. |
| **4. Discussion and conclusions:** Outcomes—Comment on the extent to which PPI influenced the study overall. Describe positive and negative effects | The patient research partners made positive and insightful contributions. It has ensured that the implementation of evidence-based OA treatment innovations from JIGSAW-E and SAMBA study had a strong patient focus. However, there were some limitations. The two patient research partners had no previous experience in research, and the complexity of the international JIGSAW-E project was challenging to comprehend, which may have influenced their overall international involvement. Also, the English language was sometimes a challenge. |
| **5. Reflections/critical perspective:**  Comment critically on the study, reflecting on the things that went well and those that did not, so others can learn from this experience | The importance and relevance for people with OA was emphasised by the patient research partners, and their early involvement and contribution added value to the project. This highlights the importance of early and sustained public involvement in these types of implementation project studies. Future studies should provide more pre-training to patient research partners. |
